# Inclusion of spray dried plasma in diets based on different ingredient combinations increases the digestibility of energy, fiber, Ca, and P by young pigs

**DOI:** 10.1093/tas/txad031

**Published:** 2023-03-16

**Authors:** Hannah M Bailey, Joy M Campbell, Leidy J Torres-Mendoza, Natalia S Fanelli, Hans H Stein

**Affiliations:** Division of Nutritional Sciences, University of Illinois, Urbana, IL 61801, USA; APC LLC, Ankeny, IA 50021, USA; Division of Nutritional Sciences, University of Illinois, Urbana, IL 61801, USA; Division of Nutritional Sciences, University of Illinois, Urbana, IL 61801, USA; Division of Nutritional Sciences, University of Illinois, Urbana, IL 61801, USA

**Keywords:** additivity, apparent total tract digestibility, endogenous phosphorus losses, pig, spray dried plasma

## Abstract

An experiment was conducted to test the hypothesis that inclusion of spray dried plasma (SDP) in diets increases apparent total tract digestibility (ATTD) and/or the standardized total tract digestibility (STTD) of gross energy (GE) and nutrients in diets for young pigs, and that ATTD of energy and nutrients or STTD of P in individual ingredients are additive in diets containing SDP. Eighty barrows (body weight: 9.30 ± 0.97 kg) were housed in individual metabolism crates and allotted to 1 of 10 diets in a randomized complete block design with 8 replicate pigs per diet. Four diets were prepared without SDP and contained ingredients commonly used in the United States, Canada, the European Union, or Asia. Four additional diets were prepared by mixing 94% of the previous four diets and 6% SDP. A diet containing SDP as the sole source of P and a P-free diet were also formulated. The ATTD of GE and nutrients and the STTD of P were calculated in all diets except the P-free diet and for the four regional diets containing 6% SDP, values were also predicted from the digestibility obtained in SDP alone and the regional diets without SDP. Differences between measured and predicted values for digestibility of GE and nutrients were also calculated. An interaction was observed between SDP and region for the ATTD of soluble dietary fiber where the digestibility decreased (*P* < 0.05) for pigs fed the U.S. diet with 6% SDP compared with 0% SDP, but that was not the case for the other regional diets. There was no interaction for the ATTD of GE, N, insoluble dietary fiber (IDF), total dietary fiber (TDF), Ca, and P or the STTD of P, but the ATTD and STTD values were greater (*P* < 0.05) or tended to be greater (*P* < 0.10%) when 6% SDP was included in the diet compared with diets with 0% SDP. The ATTD of GE, IDF, TDF, and P, and the STTD of P was greater (*P* < 0.05) for the Asia diet compared with the other diets regardless of inclusion of SDP. The measured ATTD of IDF and TDF was greater (*P* < 0.05) than the predicted values for the U.S. and European Union diets, and the measured ATTD of GE, N, Ca, and P and the STTD of P was greater (*P* < 0.05) than the predicted values for the Asia diet. In conclusion, addition of 6% SDP to a diet will increase the ATTD of energy and nutrients and the STTD of P in diets for weanling pigs, and in some cases, the measured ATTD of energy and nutrients or the STTD of P by pigs fed diets containing SDP is greater than predicted from individual ingredients.

## INTRODUCTION

Spray dried plasma (**SDP**) has been used in diets for newly weaned pigs to promote feed intake and growth due to the high palatability and high amino acid digestibility of SDP (Ermer et al., 1994; Gottlob et al., 2006; Mateo and Stein, 2007). The concentration and digestibility of energy in SDP is greater than in other protein ingredients (Gottlob et al., 2006; Wu et al., 2018). In addition, SDP is a source of completely digestible and bio-available P when fed to both pigs and poultry and SDP can be included in diets to reduce P excretion in the manure (Almeida and Stein, 2011; Munoz et al., 2020). Although the concentration of Ca in SDP is low, the apparent total tract digestibility (**ATTD**) of Ca increased when SDP was included in a corn–soybean meal diet fed to weanling pigs (Zhang et al., 2015), indicating that SDP may increase the total tract digestibility of Ca originating from other ingredients in the diet. When SDP was included in kibble fed to dogs, the digestibility of crude fiber and total dietary fiber (**TDF**) increased compared with a diet without SDP (Quigley et al., 2004), but SDP is not a source of fiber, so this indicates that SDP positively influenced the digestibility of TDF in the other dietary ingredients.

SDP is a source of highly digestible crude protein, and therefore, an increase in N digestibility by pigs or dogs fed diets with SDP can be explained (Bosi et al., 2001; Quigley et al., 2004). However, the improvement of the digestibility of other nutrients in a diet containing SDP is hypothesized to be due to the biologically active protein immunoglobulin G or other functional proteins, bioactive peptides, growth factors, or molecules that act in the intestinal tract to prevent pathogens from colonizing on the mucosal membrane, which leads to an improvement in the immunocompetence and enteric health of the animal (Pierce et al., 2005; Torrallardona, 2010). However, there are no data on the effect of SDP on total tract digestibility of energy, P, or fiber originating from other ingredients in the diet, particularly in ingredients that are used in regions such as the United States and South America (high-energy and high-protein ingredients such as corn and soybean meal), the European Union (lower energy ingredients including barley), Canada (higher fiber ingredients such as wheat and barley), and Asia (i.e., high energy and low fiber ingredients such as broken rice). The four regions account for the majority of global pig production. It is also not known if digestibility of energy or nutrients in diets containing SDP can be predicted from digestibility values in individual ingredients. Therefore, the objective of this research was to test the hypothesis that inclusion of SDP in diets increases the ATTD of energy, N, fiber, Ca, and P and the standardized total tract digestibility (**STTD**) of P originating from other ingredients in the diet, and that ATTD of energy and nutrients or STTD of P in individual ingredients are additive in diets containing SDP.

## MATERIALS AND METHODS

The Institutional Animal Care and Use Committee at the University of Illinois reviewed and approved the protocol for this experiment before animal work was initiated.

### Diets, Animals, and Experimental Design

Spray dried bovine plasma (Appetein) was sourced from APC LLC, Ankeny, IA, USA, and the same batch of SDP was used in all diets containing SDP. A common phase 1 diet containing 6% SDP and 10 phase 2 diets were formulated (Tables 1, 2, and 3). Four phase 2 diets were formulated with ingredients commonly used in commercial swine diets in four regions in the world (i.e., United States, European Union, Canada, and Asia). These diets did not contain SDP; however, four additional diets were formulated by mixing 94% of the previous four diets and 6% SDP. In addition, a diet containing SDP as the sole source of P and a P-free diet were formulated to measure basal endogenous losses of P. Vitamins and minerals were included in all diets to meet or exceed current nutritional requirement estimates of nursery pigs (NRC, 2012). A sample of the main ingredients and of all diets were collected at the time of diet mixing and used for chemical analysis.

**Table 1.** Ingredient composition of experimental diets

Item, %	Phase 1	P-free	SDP	United States		European Union		Canada		Asia	
SDP^1^	6.00	‒	20.00	‒	6.00	‒	6.00	‒	6.00	‒	6.00
Corn, ground	43.16	‒	‒	55.58	52.25	25.51	23.98	‒	‒	11.38	10.70
Wheat, ground	‒	‒	‒	‒	‒	20.00	18.80	30.00	28.20	‒	‒
Barley, ground	‒	‒	‒	‒	‒	15.00	14.10	30.00	28.20	‒	‒
Rice, ground	‒	‒	‒	‒	‒	‒	‒	‒	‒	45.00	42.30
Soybean meal	25.00	‒	‒	15.00	14.10	9.00	8.46	9.47	8.90	10.00	9.40
Fermented soybean meal	‒	‒	‒	‒	‒	‒	‒	7.00	6.58	7.00	6.58
Fish meal	‒	‒	‒	‒	‒	‒	‒	‒	‒	5.00	4.70
Whey powder	20.00	‒	‒	15.00	14.10	15.00	14.10	15.00	14.10	15.00	14.10
Soy protein concentrate	‒	‒	‒	7.00	6.58	8.00	7.52	‒	‒	‒	‒
Gelatin	‒	20.00	‒	‒	‒	‒	‒	‒	‒	‒	‒
Corn starch	‒	26.70	29.01	‒	‒	‒	‒	‒	‒	‒	‒
Solka floc	‒	4.00	4.00	‒	‒	‒	‒	‒	‒	‒	‒
Lactose	‒	20.00	20.00	‒	‒	‒	‒	‒	‒	‒	‒
Sucrose	‒	20.00	20.00	‒	‒	‒	‒	‒	‒	‒	‒
Soybean oil	3.10	4.00	4.00	4.00	3.76	4.00	3.76	5.00	4.70	4.00	3.76
Limestone, ground	1.20	0.80	1.35	1.00	0.94	1.20	1.13	1.30	1.22	0.90	0.85
Dicalcium phosphate	0.80	‒	‒	1.20	1.13	1.00	0.94	0.80	0.75	0.50	0.47
Sodium chloride	0.10	0.40	0.40	0.45	0.42	0.45	0.42	0.45	0.42	0.45	0.42
L-Lys HCl	0.29	0.70	0.12	0.37	0.35	0.45	0.42	0.58	0.55	0.40	0.38
DL-Met	0.15	0.30	0.28	0.13	0.12	0.12	0.11	0.13	0.12	0.10	0.09
L-Thr	0.05	0.40	0.02	0.10	0.09	0.10	0.09	0.10	0.09	0.10	0.09
L-Trp	‒	0.20	‒	‒	‒	‒	‒	‒	‒	‒	‒
L-His	‒	0.30	‒	‒	‒	‒	‒	‒	‒	‒	‒
L-Ile	‒	0.40	0.17	‒	‒	‒	‒	‒	‒	‒	‒
L-Leu	‒	0.75	‒	‒	‒	‒	‒	‒	‒	‒	‒
L-Val	‒	0.40	‒	‒	‒	‒	‒	‒	‒	‒	‒
Magnesium oxide	‒	0.10	0.10	‒	‒	‒	‒	‒	‒	‒	‒
Potassium carbonate	‒	0.40	0.40	‒	‒	‒	‒	‒	‒	‒	‒
Vitamin-mineral mix^2^	0.15	0.15	0.15	0.17	0.16	0.17	0.16	0.17	0.16	0.17	0.16

^1^SDP, spray dried plasma.

^2^The vitamin-micromineral premix provided the following quantities of vitamins and micro minerals per kilogram of complete diet: vitamin A as retinyl acetate, 11,136 mg; vitamin D_3_ as cholecalciferol, 2,208 mg; vitamin E as DL-alpha tocopheryl acetate, 66 mg; vitamin K as menadione dimethylprimidinol bisulfite, 1.42 mg; thiamin as thiamin mononitrate, 0.24 mg; riboflavin, 6.59 mg; pyridoxine as pyridoxine hydrochloride, 0.24 mg; vitamin B_12_, 0.03 mg; D-pantothenic acid as D-calcium pantothenate, 23.5 mg; niacin, 44.1 mg; folic acid, 1.59 mg; biotin, 0.44 mg; Cu, 20 mg as copper sulfate and copper chloride; Fe, 126 mg as ferrous sulfate; I, 1.26 mg as ethylenediamine dihydriodide; Mn, 60.2 mg as manganese sulfate; Se, 0.3 mg as sodium selenite and selenium yeast; and Zn, 125.1 mg as zinc sulfate.

**Table 2.** Analyzed nutrient composition of experimental diets (as-fed basis)^1^

Item	Phase 1	P-free	SDP	U.S.		European Union		Canada		Asia	
SDP^2^, %	6	0	20	0	6	0	6	0	6	0	6
Dry matter, %	88.30	92.71	92.18	87.34	87.53	88.36	88.31	89.05	89.61	89.26	89.25
Crude protein, %	21.15	22.83	15.91	17.10	20.63	16.88	20.38	16.77	20.24	17.36	20.94
Ash, %	5.99	1.53	3.23	5.55	5.51	5.38	5.52	5.18	5.38	5.30	5.14
Gross energy, kcal/kg	3,985	4,109	4,097	3,956	4,015	3,919	4,036	3,983	4,121	4,001	4,074
Insoluble dietary fiber, %	8.30	6.30	3.80	9.00	9.10	10.00	10.00	10.60	10.00	5.30	4.60
Soluble dietary fiber, %	1.00	ND^3^	0.10	0.30	0.10	0.70	1.00	2.20	1.00	0.10	0.10
Total dietary fiber, %	9.40	6.30	4.00	9.30	9.20	10.70	10.90	12.80	11.00	5.40	4.70
Starch, %	24.40	16.96	21.73	33.06	29.18	31.22	29.42	29.75	30.62	35.54	35.50
Ca, %	0.89	0.33	0.57	0.75	0.70	0.78	0.75	0.88	0.77	0.84	0.79
P, %	0.61	0.01	0.30	0.57	0.63	0.56	0.63	0.59	0.61	0.55	0.61
Indispensable amino acids, %											
Arg	1.22	1.47	0.87	1.06	1.17	0.98	1.17	0.89	1.27	1.13	1.30
His	0.55	0.31	0.47	0.44	0.54	0.41	0.52	0.37	0.54	0.43	0.53
Ile	0.90	0.49	0.61	0.79	0.86	0.74	0.83	0.69	0.90	0.81	0.88
Leu	1.85	1.33	1.48	1.54	1.84	1.37	1.69	1.20	1.73	1.45	1.76
Lys	1.61	1.20	1.53	1.29	1.58	1.34	1.73	1.20	1.82	1.36	1.78
Met	0.44	0.42	0.39	0.41	0.41	0.38	0.37	0.37	0.41	0.44	0.44
Phe	1.03	0.41	0.84	0.86	1.00	0.82	0.99	0.76	1.09	0.86	1.02
Thr	1.03	3.32	1.04	0.80	1.00	0.74	0.95	0.75	1.04	0.80	1.03
Trp	0.34	0.27	0.33	0.24	0.30	0.23	0.32	0.22	0.29	0.25	0.32
Val	1.16	0.68	1.09	0.88	1.11	0.84	1.09	0.79	1.17	0.94	1.18
Total	10.13	9.90	8.65	8.31	9.81	7.85	9.66	7.24	10.26	8.47	10.24
Dispensable amino acids, %											
Ala	1.03	1.63	0.78	0.85	1.02	0.76	0.93	0.67	0.95	0.93	1.04
Asp	2.12	1.13	1.62	1.79	2.05	1.60	1.92	1.42	2.05	1.83	2.11
Cys	0.43	0.03	0.54	0.30	0.43	0.30	0.44	0.30	0.52	0.30	0.43
Glu	3.53	1.96	2.19	3.13	3.45	3.30	3.76	3.34	4.13	3.02	3.38
Gly	0.80	4.45	0.58	0.67	0.77	0.67	0.79	0.64	0.87	0.87	0.90
Pro	1.26	2.58	0.88	1.08	1.21	1.19	1.34	1.24	1.48	1.08	1.16
Ser	1.00	0.61	0.94	0.77	0.98	0.73	0.95	0.68	1.01	0.78	0.96
Tyr	0.79	0.27	0.77	0.65	0.75	0.59	0.75	0.54	0.80	0.70	0.90
Total	10.96	12.66	8.30	9.24	10.66	9.14	10.88	8.83	11.81	9.51	10.88
Total amino acids, %	21.09	22.56	16.95	17.55	20.47	16.99	20.54	16.07	22.07	17.98	21.12

^1^The phase 1 SDP diet was formulated based on the requirements of 5 to 7 kg pigs, and all other diets, except the P-free and SDP diet, were formulated based on the requirements of 7 to 11 kg pigs (NRC, 2012).

^2^SDP, spray dried plasma.

^3^ND, not detected.

**Table 3.** Analyzed nutrient composition of ingredients (as-fed basis)

Item	SDP^1^	Corn	Soybean meal	Fermented soybean meal	Wheat	Barley	Soy protein concentrate	Rice	Fish meal	Whey powder	Gelatin
Dry matter, %	91.71	84.81	88.22	89.72	87.53	87.77	88.63	87.69	91.93	90.92	89.94
Crude protein, %	79.62	6.94	45.15	51.63	11.38	10.56	59.46	7.40	62.78	12.38	100.87
Ash, %	7.42	1.06	6.59	7.40	1.55	1.71	6.52	0.50	19.80	8.25	0.08
Gross energy, kcal/kg	4,826	3,721	4,127	4,337	3,841	3,863	4,238	3,746	4,416	3,654	4,656
Insoluble dietary fiber, %	4.80	8.80	15.10	9.10	11.00	14.00	17.20	2.00	4.00	0.20	0.10
Soluble dietary fiber, %	ND^2^	0.30	1.30	5.10	0.30	4.50	0.70	ND	0.50	ND	ND
Total dietary fiber, %	4.80	9.20	16.40	14.10	11.30	18.60	17.90	2.00	4.50	0.20	0.10
Starch, %	NA^3^	57.88	NA	NA	55.42	39.55	NA	69.18	NA	NA	NA
Ca, %	0.13	0.01	0.29	0.55	0.03	0.11	0.45	0.01	5.31	0.75	ND
P, %	1.58	0.24	0.63	0.78	0.35	0.69	0.80	0.12	3.28	0.95	ND
Indispensable amino acids, %											
Arg	4.52	0.35	3.25	3.35	0.57	0.57	4.39	0.58	3.56	0.28	7.82
His	2.44	0.21	1.19	1.30	0.27	0.25	1.59	0.18	1.42	0.22	0.90
Ile	2.52	0.27	2.18	2.50	0.40	0.39	2.95	0.32	2.55	0.73	1.32
Leu	7.44	0.86	3.53	3.98	0.77	0.74	4.76	0.62	4.19	1.20	2.81
Lys	7.11	0.25	2.92	2.93	0.36	0.43	3.94	0.28	4.47	1.04	3.94
Met	0.94	0.16	0.62	0.73	0.19	0.18	0.86	0.19	1.67	0.21	0.93
Phe	4.18	0.36	2.36	2.62	0.51	0.56	3.16	0.40	2.31	0.37	1.94
Thr	5.09	0.27	1.76	2.01	0.33	0.37	2.43	0.26	2.38	0.77	1.66
Trp	1.52	0.06	0.65	0.72	0.12	0.11	0.82	0.09	0.60	0.22	0.02
Val	5.65	0.37	2.32	2.66	0.51	0.55	3.02	0.45	2.98	0.69	2.32
Total	41.41	3.16	20.78	22.80	4.03	4.15	27.92	3.37	26.13	5.73	23.64
Dispensable amino acids, %											
Ala	3.83	0.53	1.97	2.31	0.43	0.44	2.64	0.42	3.94	0.58	8.20
Asp	7.83	0.50	5.10	5.72	0.64	0.69	6.91	0.66	5.27	1.24	5.60
Cys	2.60	0.16	0.65	0.76	0.28	0.23	0.87	0.17	0.49	0.29	0.12
Glu	10.78	1.33	8.22	9.08	3.07	2.53	11.29	1.36	7.72	2.03	9.80
Gly	2.74	0.29	1.92	2.28	0.48	0.45	2.54	0.33	4.68	0.24	20.80
Pro	4.07	0.62	2.43	2.72	1.06	1.13	3.17	0.37	3.01	0.70	12.31
Ser	4.55	0.34	1.99	2.26	0.47	0.42	3.06	0.36	2.01	0.49	2.95
Tyr	3.82	0.27	1.70	1.83	0.34	0.30	2.16	0.25	1.80	0.28	0.76
Total	40.22	4.04	23.98	26.96	6.77	6.19	32.64	3.92	28.92	5.85	60.54
Total amino acids, %	81.63	7.20	44.76	49.76	10.80	10.34	60.56	7.29	55.05	11.58	84.18

^1^SDP, spray dried plasma.

^2^ND, not detected.

^3^NA, not analyzed.

Eighty weanling barrows (body weight of 6.53 ± 0.59 kg) were allotted to a randomized complete block design with two weaning date blocks of 40 pigs. Within each block, the 40 pigs were housed in groups of five and fed the phase 1 diet for 2 wk immediately postweaning. After 2 wk, when pigs had a body weight of 9.30 ± 0.97 kg, they were housed individually in metabolism crates that were equipped with a feeder, a nipple drinker, a fully slatted floor, a screen floor, and a urine tray, which allowed for the total, but separate, collection of feces and urine. The 80 pigs were divided into two blocks of 40 pigs and in each block, pigs were randomly allotted to the 10 experimental diets, resulting in 4 replicate pigs per diet per block for a total of 8 replicate pigs per diet.

### Feeding and Sample Collection

All pigs were provided feed at a daily level of 3.3 times the maintenance energy requirement (i.e., 197 kcal/kg body weight^0.60^; NRC, 2012). The daily feed allowance was provided in two equal meals at 0700 and 1600 hours and feed consumption was recorded daily. Water was available at all times throughout the experiment. All pigs were fed experimental diets for 11 d, with the initial 5 d of the experiment being the adaptation period to the diet followed by 4 d of total fecal and urine collection according to the marker-to-marker procedure (Adeola, 2001). Fecal collection began when the first marker (i.e., indigo carmine), fed in the morning meal on day 6, appeared in the feces, and ceased when the second marker (i.e., ferric oxide), fed in the morning meal on day 10, appeared in the feces. Orts were collected and weighed daily to determine feed intake.

All pigs were weighed at the beginning of the experiment (at weaning), prior to moving into metabolism crates, and at the end of both the adaptation and collection period. During the collection period, feces were collected twice daily and stored at −20 °C as soon as collected, and urine was collected over a preservative of 50 mL of 6 N HCl in buckets placed under the metabolism crates. The urine buckets were emptied once daily, the weight of the collected urine was recorded, and 20% was stored at −20 °C. At the conclusion of the experiment, urine samples were thawed and mixed within animal and diet and 2 subsamples were collected. One subsample was lyophilized and the other subsample was stored at −20 °C until analysis for N. At the end of the experiment, pigs had an average body weight of 12.59 ± 1.66 kg

### Chemical Analysis

All collected fecal samples were dried at 65 °C in a forced air oven (Metalab Equipment Corp., Hicksville, NY, USA) and finely ground using a 500G stainless steel mill grinder (RRH, Zhejiang, China) prior to chemical analysis. The lyophilized urine samples, fecal samples, and all diet and ingredient samples were analyzed in duplicate for concentrations of gross energy (GE) using an isoperibol bomb calorimeter (Model 6400, Parr Instruments, Moline, IL, USA). Diet and fecal samples and the urine samples that were not lyophilized were analyzed for N using the Kjeldahl method (method 984.13; AOAC Int., 2019) on a KjeltecTM 8400 (FOSS Inc., Eden Prairie, MN, USA) with subsequent calculation of crude protein by a conversion factor of 6.25. Fecal, diet, and ingredient samples were also analyzed in duplicate for dry matter by oven drying at 135 °C for 2 h (method 930.15, AOAC Int., 2019), and for dry ash at 600 °C for 3 h (method 942.05; AOAC Int., 2019). Insoluble dietary fiber (**IDF**) and soluble dietary fiber (**SDF**) were analyzed according to method 991.43 (AOAC Int., 2019) using the Ankom TDF Analyzer (Ankom Technology, Macedon, NY, USA). Fecal, diet, and ingredient samples were also analyzed for Ca and P using inductively coupled plasma-optical emission spectrometry (ICP-OES; Avio 200, PerkinElmer, Waltham, MA, USA). Sample preparation included dry ash (method 985.01 A, B and C; AOAC Int., 2019) and wet digestion with nitric acid (method 3050 B; U.S. Environmental Protection Agency, 2000). All diet and ingredient samples were analyzed for amino acids on a Hitachi Amino Acid Analyzer, Model No. L8800 (Hitachi High Technologies America, Inc.; Pleasanton, CA, USA) using ninhydrin for postcolumn derivatization and norleucine as the internal standard [method 982.30 E (a, b, c); AOAC Int., 2019] and for total starch using the glucoamylase procedure (method 979.10; AOAC Int., 2019).

### Calculations

Using the direct procedure, the ATTD of GE, N, SDF, IDF, TDF, Ca, and P in the SDP diet and all phase 2 diets, except the P-free diet, was calculated (Almeida and Stein, 2010; NRC, 2012). The digestible energy (**DE**) in all diets were calculated by subtracting the GE in feces from the intake of GE in the diet, and the metabolizable energy (**ME**) in all diets were calculated by subtracting the GE in feces and urine from the intake of GE in the diet (NRC, 2012). The basal endogenous P loss (**EPL**; mg/kg of dry matter intake; **DMI**) was determined from pigs fed the P-free and from pigs fed the SDP diet, and the STTD of P was calculated in each diet except the P-free diet by correcting the ATTD values for EPL (Almeida and Stein, 2010; NRC, 2012).

The predicted ATTD of GE in the regional diets containing SDP was calculated according to the following equation (Stein et al., 2005; Xue et al., 2014):


$$\begin{array}{ll} ATTD_{GE}= & \left[(GE_{SDP}\times ATTD_{SDP})+\left(GE_{D}\times ATTD_{D}\right)\right]\\ & /\left(GE_{SDP}+GE_{D}\right), \end{array}$$

where ATTD_GE_ is the predicted ATTD for GE (%) in the regional diet with SDP; GE_SDP_ and GE_D_ are the concentrations (%) of GE contributed by SDP and regional diets without SDP, respectively, which were calculated by multiplying the concentration of GE (%) in that ingredient by the proportion (%) of that ingredient in the regional diet with SDP; and ATTD_SDP_ and ATTD_D_ is the measured ATTD (%) for GE in SDP and the regional diets without SDP, respectively. The predicted ATTD of nutrients and STTD of P in regional diets containing SDP were also calculated using this equation.

The retention of N and the biological value of protein were also calculated (Pedersen et al., 2007; Rojas and Stein, 2013).

### Statistical Analysis

Normality of residuals was verified and outliers were identified using the UNIVARIATE and BOXPLOT procedures in SAS (9.4 version, SAS Inst. Inc., Cary, NC, USA), respectively. Outliers were removed if the value deviated from the 1st or 3rd quartiles by more than three times the interquartile range (Tukey, 1977). Data were analyzed as a 2 × 4 factorial arrangement of treatments using the MIXED procedure of SAS (SAS Institute Inc.) with two levels of SDP and four regions. The pig was the experimental unit for all analyses. The model included dietary concentration of SDP, region, and the interaction between SDP and region as fixed effects and block and replicate within block as random effects. Data from pigs fed the P-free and SDP diet were compared using the MIXED procedure of SAS (SAS Institute Inc.) where the statistical model included diet as the fixed effect and block and replicate within block as random effects. Treatment means were calculated using the LSMEANS statement in SAS, and if significant, means were separated using the PDIFF option in the MIXED procedure.

Additivity of ATTD and STTD values in the four regional diets was analyzed by comparing measured and predicted values using a *t* test; the null hypothesis that the difference between measured and predicted values for ATTD and STTD was equal to 0 was tested (She et al., 2018). Statistical differences were established at *P* ≤ 0.05, and 0.05 ˂ *P* ≤ 0.10 was considered a trend.

## RESULTS

The ATTD of GE in SDP was 94.3% and the ATTD of N was 94.1% (Table 4). For Ca and P, the ATTD values in SDP were 77.2% and 90.6%, respectively, and the STTD of P in SDP was 96.5%. The ATTD of dry matter was greater (*P* < 0.05) for the P-free diet than the SDP diet (Table 5). However, feed intake, P intake, fecal excretion, P in feces, fecal P excretion, and basal EPL, were greater (*P* < 0.05) for pigs fed the diet with SDP as the sole source of P compared with the P-free diet.

**Table 4.** Apparent total tract digestibility (ATTD) of gross energy (GE), Ca, P, and fiber, and standardized total tract digestibility (STTD) of P in spray dried plasma^1,2^

Item, %	Spray dried plasma
ATTD	
Gross energy	94.3
N	94.1
Ca	77.2
P	90.6
Total dietary fiber	41.5
Insoluble dietary fiber	41.2
Soluble dietary fiber	−4.9
STTD^2^	
P	96.5

^1^Data are least squares means of eight observations.

^2^The STTD of P in spray dried plasma was calculated by correcting ATTD of P for basal endogenous P loss that was obtained from pigs fed the P-free diet = 193 mg/kg dry matter intake.

**Table 5.** Basal endogenous P loss (EPL), apparent total tract digestibility (ATTD) of dry matter and P, and standardized total tract digestibility (STTD) of P in a P-free diet and diet with spray dried plasma (SDP) as the sole source of P fed to pigs^1^

Item	P-free	SDP	Pooled SEM	*P*-value
Feed intake, g/d	482.0^b^	577.1^a^	18.36	0.017
P intake, g/d	0.05^b^	1.74^a^	0.027	<0.001
Fecal excretion, g/d	16.96^b^	28.92^a^	0.798	<0.001
P in feces, %	0.45^b^	0.71^a^	0.023	<0.001
Fecal P excretion, g/d	0.06^b^	0.16^a^	0.006	<0.001
ATTD of dry matter, %	97.2^a^	95.9^b^	0.08	<0.001
Basal EPL, mg/kg DMI	192.6^b^	370.3^a^	10.12	<0.001

^1^Data are least square means of six to eight observations.

^a,b^Means within a row lacking a common superscript letter differ (*P* < 0.05).

Pigs fed the European Union diet with 6% SDP had greater (*P* < 0.05) feed intake than pigs fed the European Union diet without SDP (Table 6), but feed intake of pigs fed the other regional diets was not increased if 6% SDP was included (interaction, *P* < 0.05). Intake of GE was greater (*P* < 0.05) for pigs fed the European Union diet with 6% SDP compared with pigs fed the same diet without SDP, but there was no difference in GE intake among pigs fed the other diets without or with 6% SDP (interaction, *P* < 0.05). The intake of Ca, TDF, and IDF was increased (*P* < 0.05) for pigs fed the European Union diet with 6% SDP compared with pigs fed the European Union diet without SDP, but intake of Ca, TDF, and IDF was less (*P* < 0.05) for pigs fed the Canada or Asia diet with 6% SDP than for pigs fed the diets with no SDP, but the intake of those nutrients did not change if SDP was included in the U.S. diet (interaction, *P* < 0.05). In contrast, the intake of P increased (*P* < 0.05) for pigs fed the United States, European Union, or Asia diet with 6% SDP compared with pigs fed those diets without SDP, but that was not the case for pigs fed the Canada diet (interaction, *P* < 0.05). Intake of SDF decreased (*P* < 0.05) for pigs fed the U.S. or Canada diets with 6% SDP compared with pigs fed those diets with no SDP, and increased (*P* < 0.05) for pigs fed the European Union diet with 6% SDP compared with pigs fed the European Union diet without SDP (interaction, *P* < 0.05).

**Table 6.** Nutrient intakes and outputs by pigs fed regional diets without or with spray dried plasma (SDP; as-fed basis)

Item	U.S.		European Union		Canada		Asia		Pooled SEM	*P*-value		
SDP, %	0	6	0	6	0	6	0	6		SDP	Region	Interaction
Intake												
Feed, g/d	617^b^	633^a,b^	609^b^	674^a^	662^a^	632^a,b^	638^a,b^	631^a,b^	22.2	0.297	0.522	0.022
GE^1^ intake, Mcal/d	2.44^c,d^	2.54^b,c,d^	2.39^d^	2.72^a^	2.64^a,b^	2.61^a,b.c^	2.55^a,b,c,d^	2.57^a,b,c^	0.089	0.019	0.245	0.023
Ca, g/d	4.64^e,f^	4.46^f^	4.74^d,e,f^	5.09^b,c^	5.80^a^	4.87^c,d,e^	5.36^b^	5.01^c,d^	0.174	0.002	<0.001	<0.001
P, g/d	3.52^c^	3.98^b^	3.41^c^	4.24^a^	3.91^b^	3.86^b^	3.54^c^	3.83^b^	0.130	<0.001	0.139	<0.001
TDF^2^, g/d	57.4^d^	58.3^d^	65.1^c^	73.5^b^	84.7^a^	69.6^b,c^	34.5^e^	29.7^f^	2.18	0.026	<0.001	<0.001
IDF^3^, g/d	55.5^e^	57.6^d,e^	60.9^c,d^	67.4^a,b^	70.2^a^	63.3^b,c^	33.8^f^	29.0^g^	2.02	0.489	<0.001	<0.001
SDF^4^, g/d	1.85^d^	0.63^e^	4.26^c^	6.74^b^	14.56^a^	6.32^b^	0.64^e^	0.63^e^	0.195	<0.001	<0.001	<0.001
Output												
Fecal, g/d	67.2^y^	65.5^y^	69.8^y^	72.6^y^	85.2^x^	71.1^y^	47.5^z^	39.6^z^	4.18	0.031	<0.001	0.075
Urine, kg/d	1.86	2.53	1.83	2.28	2.10	3.08	2.54	2.70	0.42	0.059	0.433	0.790
GE in feces, kcal/kg	4,795	4,818	4,740	4,710	4,690	4,666	4,647	4,629	37.4	0.597	<0.001	0.832
GE output, kcal/d	322	316	331	342	399	341	221	183	21.7	0.052	<0.001	0.164
GE in urine, kcal/kg	51.6	46.9	44.1	41.1	46.2	39.5	36.3	38.1	6.48	0.425	0.213	0.897
Urine GE output, kcal/d	73.6	103.6	68.6	98.7	85.9	107.9	93.0	88.8	9.01	0.002	0.450	0.148
Ca in feces, %	1.96	1.98	2.04	1.94	2.04	1.91	3.57	3.16	0.142	0.136	<0.001	0.482
Ca output, g/d	1.30	1.29	1.42	1.24	1.74	1.35	1.71	1.24	0.124	0.002	0.100	0.171
P in feces, %	2.11	2.14	2.02	2.01	1.95	1.97	2.60	2.66	0.083	0.691	<0.001	0.985
P output, g/d	1.12	1.12	1.12	1.03	1.33	1.11	1.00	0.84	0.080	0.030	0.002	0.534
TDF in feces, %	31.1	30.5	34.3	33.3	36.7	37.2	20.6	20.7	1.06	0.662	<0.001	0.846
TDF output, g/d	20.8	19.9	23.9	23.8	31.3	26.4	9.8	8.1	1.21	0.013	<0.001	0.103
IDF in feces, %	27.6	27.5	31.9	30.5	34.2	34.6	16.9	18.2	0.89	0.955	<0.001	0.384
IDF output, g/d	18.6	18.0	22.1	21.9	29.1	25.3	8.5	7.1	1.10	0.029	<0.001	0.253
SDF in feces, %	3.41	3.08	2.46	2.78	2.89	2.60	2.26	2.48	0.300	0.905	0.030	0.571
SDF output, g/d	2.27	1.96	1.72	1.96	2.47	1.84	1.31	0.97	0.202	0.073	<0.001	0.199

^1^GE, gross energy.

^2^TDF, total dietary fiber.

^3^IDF, insoluble dietary fiber.

^4^SDF, soluble dietary fiber.

^a,b,c,d,e,f,g^Means within a row lacking a common superscript letter differ (*P* < 0.05).

^x,y,z^Means within a row lacking a common superscript letter differ (*P* < 0.10).

Pigs fed the Canada diet with no SDP had a tendency for greater (*P* < 0.10) fecal output than pigs fed the Canada diet with 6% SDP, but the fecal output of pigs fed the other regional diets was not impacted by the level of SDP in the diet (interaction, *P* < 0.05). There was no interaction between inclusion of SDP and region for the output of urine, GE, Ca, P, IDF, SDF, and TDF. The DE in diets with 6% SDP was increased (*P* < 0.05) compared with diets without SDP, but the increase in DE of the diet was greater when 6% SDP was included in the Asia diet compared with the other diets (interaction, *P* < 0.05), and DE was greater (*P* < 0.05) for the Canada diet than the U.S. and European Union diet when 6% SDP was included. The ME in the European Union, Canada, and Asia diet increased (*P* < 0.05) when 6% SDP was included compared with diets without SDP, but ME in the United States diet with 6% SDP was not different from the ME in the diet without SDP (interaction, *P* < 0.05).

There was no interaction between inclusion of SDP and region for the ATTD of GE, Ca, P, TDF, or IDF and the STTD of P (Table 7), however, an interaction between SDP and region was observed for the ATTD of SDF where digestibility decreased (*P* < 0.05) for pigs fed the U.S. diet with 6% SDP compared with no SDP, but the ATTD of SDF was not affected by SDP inclusion for the other regional diets. The ATTD of DM, GE, Ca, P, and IDF and the STTD of P was greater (*P* < 0.05) when 6% SDP was included in the diet compared with 0% SDP regardless of region, and the ATTD of TDF tended to increase (*P* < 0.10) for pigs fed a diet with 6% SDP compared with a diet without SDP. The ATTD of DM, GE, P, TDF, and IDF and the STTD of P was greater (*P* < 0.05) for the Asia diet compared with the other diets regardless of inclusion of SDP.

**Table 7.** Concentrations of digestible energy (DE) and metabolizable energy (ME), apparent total tract digestibility (ATTD) of gross energy (GE), Ca, P, and fiber, and standardized total tract digestibility (STTD) of P in regional diets without or with spray dried plasma (SDP; as-fed basis)

Item	U.S.		European Union		Canada		Asia		Pooled SEM	*P*-value		
SDP, %	0	6	0	6	0	6	0	6		SDP	Region	Interaction
DE in diet, kcal/kg	3,435^e^	3,519^d^	3,376^e^	3,531^d^	3,381^e^	3,594^c^	3,655^b^	3,784^a^	26.9	<0.001	<0.001	0.039
ME in diet, kcal/kg	3,314^e,f^	3,338^d,e^	3,265^e,f^	3,385^c,d^	3,251^f^	3,423^c^	3,509^b^	3,627^a^	29.8	<0.001	<0.001	0.040
ATTD, %												
GE	86.8	87.6	86.2	87.5	84.9	87.2	91.4	92.9	0.67	<0.001	<0.001	0.568
Ca	71.6	70.9	70.0	72.5	70.9	74.5	68.2	75.3	2.1	0.041	0.894	0.303
P	68.0	71.9	67.2	72.4	67.7	71.8	72.0	78.2	1.60	<0.001	<0.001	0.832
TDF^1^	63.9	66.2	63.4	67.7	63.1	62.1	71.7	72.6	1.35	0.085	<0.001	0.214
IDF^2^	66.7	68.9	63.7	67.7	58.6	61.2	75.0	75.4	1.34	0.018	<0.001	0.558
SDF^3^	−22.3^b^	−191.0^d^	59.5^a^	70.8^a^	83.3^a^	71.0^a^	−102.8^c^	−54.9^b,c^	22.7	0.063	<0.001	<0.001
STTD^4^, %												
P	71.0	74.5	70.2	75.2	70.6	74.7	75.1	81.0	1.60	<0.001	<0.001	0.848

^1^TDF, total dietary fiber.

^2^IDF, insoluble dietary fiber.

^3^SDF, soluble dietary fiber.

^4^The STTD of P in diets was calculated by correcting ATTD of P for basal endogenous P loss that was obtained from pigs fed the P-free diet = 193 mg/kg dry matter intake.

^a,b,c,d,e,f^Means within a row lacking a common superscript letter differ (*P* < 0.05).

Intake of N was greater in all regional diets if 6% SDP was included (Table 8) compared with no SDP in the diet, but the increase was greater for the United States and European Union diets than for the Canada and Asia diets (interaction, *P* < 0.05). Pigs fed diets with 6% SDP had greater N retention during the 4-d collection period than pigs fed diets without SDP, but the increase tended to be greater for pigs fed the European Union or Asia diets than for pigs fed the other diets (interaction, *P* < 0.10). Pigs fed a diet with 6% SDP had greater (*P* < 0.05) ATTD of N compared with pigs fed a diet without SDP regardless of region, and pigs fed the Asia diet had greater (*P* < 0.05) ATTD of N than pigs fed the Canada or European Union diet regardless of SDP inclusion. There was a tendency for the biological value of N to increase (*P* < 0.10) for pigs fed the United States diet compared with the Asia diet regardless of SDP inclusion.

**Table 8.** Nitrogen balance of pigs fed regional diets without or with spray dried plasma (SDP) during a 4-d collection period (as-fed basis)

Item	U.S.		European Union		Canada		Asia		Pooled SEM	*P*-value		
SDP, %	0	6	0	6	0	6	0	6		SDP	Region	Interaction
N intake, g/4 d	67.5^c,d^	83.6^a,b^	65.7^d^	88.0^a^	71.0^c^	81.9^b^	70.9^c^	84.6^a,b^	2.63	<0.001	0.681	0.018
N output in feces, g/4 d	11.4^y,z^	11.9^x,y^	11.7^y,z^	12.8^x,y^	13.6^x^	11.6^x,y,z^	11.5^y,z^	9.9^z^	0.74	0.295	0.032	0.057
N output in urine, g/4 d	6.8	10.9	7.5	11.6	8.8	11.4	10.3	12.1	1.10	<0.001	0.018	0.292
ATTD^1^ of N, %	83.1	85.8	82.3	85.5	80.7	85.1	83.8	88.3	0.81	<0.001	0.003	0.578
N retention, g/4 d	51.3^y^	60.5^w,x^	46.6^z^	63.6^w^	48.0^y,z^	58.4^x^	49.1^y,z^	62.6^w,x^	2.14	<0.001	0.269	0.073
N retention, %	73.0	72.3	70.8	73.3	67.5	71.0	69.3	74.1	1.73	0.003	0.020	0.101
Biological value^2^, %	87.8	84.3	85.9	84.6	84.4	83.4	82.8	84.0	1.79	0.152	0.089	0.250

^1^ATTD, apparent total tract digestibility.

^2^Biological value was calculated as (N retained/ [N intake—N output in feces]) × 100 (Rojas and Stein, 2013).

^a,b,c,d^Means within a row lacking a common superscript letter differ (*P* < 0.05).

^w,x,y,z^Means within a row lacking a common superscript letter differ (*P* < 0.10).

The measured ATTD of GE was greater (*P* < 0.05) than the predicted value in the Canada and Asia diet with SDP (Table 9), and the measured value for ATTD of N, Ca, and P and the STTD of P was greater (*P* < 0.05) than the predicted value in the Asia diet with SDP. The measured ATTD of TDF was greater (*P* < 0.05) than predicted in the United States and European Union diet with SDP, and the measured ATTD of IDF was greater (*P* < 0.05) than the predicted value in the United States, European Union, and Canada diet with SDP. The measured ATTD of SDF was greater (*P* < 0.05) than predicted in the European Union and Asia diet with SDP, but the measured value was less (*P* < 0.05) than predicted in the United States and Canada diet with SDP. When calculating the overall effect of SDP in diets fed to pigs, it was observed that the measured ATTD of GE, N, Ca, P, TDF, and IDF, but not the ATTD of SDF or STTD of P, was greater (*P* < 0.05) than predicted values when SDP was included in the diet.

**Table 9.** Differences^1^ between measured and predicted apparent total tract digestibility (ATTD) values for gross energy, N, Ca, P, and fiber and for standardized total tract digestibility (STTD) of P in regional diets with spray dried plasma from the United States, European Union, Canada, and Asia^2,3^

Item	U.S.	European Union	Canada	Asia	Overall^4^
ATTD, %					
Gross energy	0.14	0.71	1.65*	1.29*	3.36*
N	0.12	0.47	1.29	2.20*	2.36*
Ca	−0.83	2.45	3.56*	6.95*	3.10*
P	0.44	1.79	0.92	3.31*	2.77*
TDF	2.76*	4.89*	−0.52	2.41	3.48*
IDF	2.89*	4.70*	3.10*	2.19	5.56*
SDF	−167.60*	9.44*	−13.06*	71.05*	−0.82
STTD, %					
P	−0.26	1.03	0.41	2.62*	1.64

^1^Difference is calculated by subtracting predicted ATTD of gross energy, N, Ca, or P from measured value. Likewise, the difference between predicted and measured values for STTD of P were calculated.

^2^Data are least square means of six to eight observations, except for the calculation of overall which are least square means of 30 to 32 observations.

^3^*P ≤ 0.05.

^4^Overall was calculated as sum of the differences from all the diets with spray dried plasma.

## DISCUSSION

In the present experiment, an inclusion level of SDP of 6% was chosen because it is a fairly typical inclusion rate in countries where SDP is included in phase 1 diets for weanling pigs. By mixing the basal diets and SDP in a 94:6 ratio, it was possible to calculate the impact of SDP on the ATTD of energy and nutrients in the four regional diets using the substitution procedure. Whereas some ingredients are typically used in only certain regions of the world, it is recognized that diets for pigs are widely diverse in all regions of the world and the diets used in this experiment for the four regions are only examples of diets in these regions, and it is not the intend to infer that these are the only diets used in each region.

When comparing the digestibility results from the current experiment with published data on the digestibility of GE and nutrients in SDP, the ATTD of GE agrees with values observed by Wu et al. (2018) and the ATTD and STTD of P in SDP agrees with values by Almeida et al. (2011). The ATTD of N in SDP has not been reported, but the apparent ileal digestibility of N in SDP was 82% to 85% (Almeida et al., 2013; Jeong et al., 2016), which is less than the 94.1% ATTD of N observed in the current experiment indicating that there may have been absorption of N from the hindgut (Stein, 2017). To our knowledge, the ATTD of Ca, TDF, IDF, or SDF in SDP has not been reported because the concentration of these nutrients in SDP is negligible. In comparison with blood meal, the ATTD and STTD of P in SDP is greater (Almeida et al., 2011), but the ATTD of Ca and TDF in other blood products has not been reported.

The digestibility of GE in rice is greater compared with that in corn, wheat, or barley (Cervantes-Pahm et al., 2014), which likely is the reason for the greater ATTD of GE and greater concentrations of DE and ME in the Asia diets compared with the other diets. The negative values that were observed for the ATTD of SDF in both the Asia and U.S. diets are a result of microbial material in the feces that was analyzed as TDF, and negative values for the ATTD of SDF have been previously reported (Jørgensen et al., 1996; Wilfart et al., 2007; Cervantes-Pahm et al., 2013). The amount of TDF analyzed in the Canada and European Union diets was greater than in the United States and Asia diets because of the inclusion of wheat and barley in these diets. Wheat and barley have greater concentrations of soluble fibers, specifically β-glucans and arabinoxylans, compared with corn and rice, which increases viscosity of digesta in the small intestine and leads to a reduction in nutrient digestion and absorption due to decreased interactions between nutrients and digestive enzymes (Cervantes-Pahm et al., 2014). Therefore, the increased ATTD of P, TDF, and IDF in the Asia diet compared with the other diets may be due to the reduced concentration of SDF in this diet.

The observation that if 6% SDP was included in the diet, the ATTD of energy and nutrients and STTD of P increased, regardless of diet formulation, agrees with data from Zhang et al. (2015) and is a result of the high digestibility of energy and nutrients in SDP compared with many other ingredients. Specifically the high ATTD and STTD of P in SDP is the reason for the increase in ATTD and STTD of P in diets with SDP (Almeida et al., 2011; Munoz et al., 2020). Additionally, the beneficial effect of SDP on ATTD of nutrients is suggested to be related to increased palatability in diets as well as the effects of immunoglobulin G, which protects intestinal mucosal surfaces from pathogen colonization, therefore increasing digestive enzyme activities (Ermer et al., 1994; Pierce et al., 2005).

To determine the STTD of P, values for the ATTD of P are corrected for basal EPL, which can be determined by feeding a P-free diet (Petersen and Stein, 2006). Values for the basal EPL that were determined by feeding a P-free diet in this experiment (193 mg/kg DMI) agree with the average for EPL from a large number of experiments of 190 mg/kg DMI (NRC, 2012). Basal EPL were also measured in this experiment by feeding a diet with 20% SDP as the only source of P, which is a common method used in Brazil because P in SDP is believed to be 100% digestible, and therefore, all P excreted in feces from pigs fed a diet with SDP is believed to be of endogenous origin (Bünzen et al., 2012; Alves et al., 2016). However, basal EPL measured from the SDP diet (370 mg/kg DMI) was greater than basal EPL measured from the P-free diet, which indicates that the absorption of P from SDP is not 100%. Excretion of P in feces and ATTD of P in diets in which all P was from SDP was greater in a diet with 30% SDP compared with a diet with 10% SDP (Alves et al., 2016), further indicating that the digestibility of P in SDP is high, but not 100%. As a consequence, estimating EPL from a diet containing SDP results in an overestimation of basal EPL. To correct this overestimation, the indigestible fraction needs to be considered in the calculation of basal EPL (Alves et al., 2016), or the inclusion of SDP in the diet should be reduced to limit undigested dietary P in the feces.

The observed increase in ATTD of N in diets containing SDP compared with diets without SDP agrees with data from mice and pigs (Thomson et al., 1995; Pan et al., 2019). An increase in ATTD of dry matter and N was also observed if SDP was included in a diet fed to pigs compared with a diet containing wheat gluten (Pendergraft et al., 1993). The improvement in N digestibility and retention by pigs fed a SDP-containing diet is due to the high bioavailability and digestibility of amino acids in SDP (Mateo and Stein, 2007; Almeida et al., 2013). The improvement in dietary protein utilization by pigs fed a diet with SDP may also be due to reduction in intestinal amino acid catabolism because of an improvement in immunocompetence (Zhang et al., 2015; Pan et al., 2019). SDP has a high concentration of immunoglobulin G and other biological active proteins, such as growth factors and peptides that acts as functional compounds in preventing the colonization of pathogenic bacteria on the surface of the intestinal mucosa and subsequently prevents overstimulation of the immune system (Torrallardona, 2010; Zhang et al., 2016). The reduced activation of the immune system in the intestinal lymphoid tissue of pigs fed a diet with SDP may improve dietary energy absorption (Nofrarías et al., 2006), and therefore, may explain the increased ATTD of GE and increased concentrations of DE and ME in diets containing 6% SDP compared with diets without SDP. The ATTD of GE in extruded kibble that included 1% to 3% SDP was observed to linearly increase with SDP inclusion (Quigley et al., 2004), further demonstrating the positive impact of SDP on digestibility of GE.

The increased ATTD of IDF and TDF in diets with SDP compared with diets without SDP agrees with results from dogs fed kibble with 1% to 3% SDP (Quigley et al., 2004) and cats fed wet pet food with 3% SDP (Rodríguez et al., 2016). However, the concentration of fiber in SDP is negligible indicating that the improvements in digestion of fiber in SDP containing diets are independent of the fiber in SDP (Quigley et al., 2004). However, microbial populations in the large intestine, specifically probiotic species such as *Lactobacillus*, may be increased in mice and pigs fed a diet with SDP (Torrallardona et al., 2003; Tran et al., 2018; Moretó et al., 2020). The density of lamina propria cells in the colon was also decreased in pigs fed a diet with SDP and a decrease in leukocytic infiltration in the intestinal mucosa was observed as well (Nofrarías et al., 2006, 2007). These observations indicate a reduced activation of the immune system and improved maintenance of the intestinal mucosa layer in pigs fed a diet containing SDP compared with pigs fed no SDP, which may have resulted in improved nutrient absorption (Campbell et al., 2019).

The mostly positive differences between measured and predicted ATTD and STTD of energy and nutrients in diets containing SDP indicate that the nutrient and energy digestibility measured in pigs fed a diet with 6% SDP was greater than what was calculated from the individual components. A difference between measured and predicted values for ATTD or STTD indicates that the quantities of digestible nutrients provided by a mixed diet is greater than the sum of digestible nutrients from the individual ingredients (She et al., 2018). It therefore appears that SDP may confer complementary effects on nutrient digestibility, which results in actual digestibility of some nutrient being greater than predicted. The reason for this observation may be that SDP in the diet reduced intestinal inflammation and maintained the integrity of the intestinal mucosa (Zhang et al., 2015; Campbell et al., 2019). The observation that diets with greater concentrations of fiber had a greater increase in ATTD of TDF than calculated for the rice-based diet may have been a result of SDP having had a prebiotic effect on the microbiome in the large intestine, thereby increasing the ATTD of fiber (Torrallardona et al., 2003). However, the rice-based diet had less fiber than the corn–soybean meal or wheat–barley based diets, which may not have provided enough substrate to generate the probiotic effect. The fact that inclusion of 6% SDP to the rice-based diet resulted in ATTD of GE, N, Ca, and P and STTD of P in the diet being greater than the predicted values indicates that the activity of brush-border enzymes may have been improved by SDP because of the improved integrity of the intestinal mucosa stimulated by SDP in the diet (Zhang et al., 2015). This effect may be greater in diets with low concentrations of fiber and reduced digesta viscosity than in diets with more fiber (Cervantes-Pahm et al., 2014). However, data for the interaction of fiber and SDP and their effect on intestinal health and nutrient digestibility are limited and further research is warranted.

In conclusion, addition of 6% SDP to a diet increased the ATTD of energy and nutrients and the STTD of P regardless of diet formulation. However, inclusion of 6% SDP to diets with low fiber ingredients had a greater complementary effect on energy and nutrient digestibility of those ingredients resulting in a greater measured digestibility of the mixed diet compared with predicted values. Therefore, the ATTD of energy and nutrients and the STTD of P for individual ingredients are not always additive in a diet containing SDP.
